# Association Between the Ki-67 Index, Tumor Size and Postoperative Structural Persistence in Non-functioning Pituitary Neuroendocrine Tumors: A Single-Center Experience

**DOI:** 10.7759/cureus.98996

**Published:** 2025-12-11

**Authors:** Carlos E Jimenez-Canizales, Juan S Salas-Henao, Gabriela Ballesteros-Oviedo, Rafael Parra-Medina, Edgar G Ordóñez-Rubiano, Johan E Vargas-Vargas, William Rojas

**Affiliations:** 1 Endocrinology, University Foundation for Health Sciences, Bogotá, COL; 2 Clinical Sciences, University of Tolima, Ibagué, COL; 3 Endocrinology, Clínica Internacional de Alta Tecnología en Cáncer (CLINALTEC), Ibagué, COL; 4 Pathology, Instituto Nacional de Cancerología, Bogotá, COL; 5 Research Institute, University Foundation for Health Sciences, Bogotá, COL; 6 Pathology, Hospital de San José, Bogotá, COL; 7 Neurosurgery, University Foundation for Health Sciences, Bogotá, COL; 8 Neurosurgery, Fundación Santa Fe de Bogotá, Bogotá, COL; 9 Family Medicine, University Foundation for Health Sciences, Bogotá, COL; 10 Endocrinology, Hospital de San José, Bogotá, COL

**Keywords:** ki-67 antigen, locoregional neoplasm recurrence, nonfunctioning adenoma, pitnets pituitary neoplasms, treatment outcome

## Abstract

Introduction

Non-functioning pituitary neuroendocrine tumors (NF-PitNETs) represent more than half of pituitary neoplasms. Features linked to greater aggressiveness include adrenocorticotropic hormone immunopositivity, plurihormonal or null-cell phenotype, elevated Ki-67 index (≥3%), and size ≥40 mm. However, evidence remains inconsistent, underscoring the need for further investigation into their biological behavior.

Methods

A retrospective study of medical records of patients with diagnoses related to pituitary pathology from 2010 to 2020 was conducted. The primary objective of this study was to evaluate the association between the Ki-67 proliferation index and tumor size with post-surgical structural persistence in patients with NF-PitNETs. The sample consisted of 81 patients who met the inclusion criteria. Clinical and paraclinical characteristics were recorded. The data were analyzed using univariate description and bivariate analysis, applying Fisher's test for the outcome of post-surgical structural persistence.

Results

The prevalence of NF-PitNETs was 58%. The most frequent symptoms were visual field disorders in 70%, followed by headache in 66%. Regarding structural persistence, a statistical association was found between the presence of Ki-67 ≥ 3% (p-value: 0.028) and tumor size ≥ 40 mm (p-value: 0.030).

Conclusion

The prevalence of NF-PitNETs is high, and the preoperative variable of tumor size (≥ 40 mm) and the Ki-67% index ≥ 3% reported in postoperative pathology were associated with greater structural persistence.

## Introduction

Non-functioning pituitary neuroendocrine tumors (NF-PitNETs) account for up to 54% of pituitary neoplasms. Furthermore, 67% to 90% of these tumors are macroadenomas, representing the leading cause of macroadenomas and the second most frequent cause of microadenomas [1]. They lack excess hormone secretion and may arise from gonadotrophic, corticotrophic, somatotrophic, lactotrophic, plurihormonal, or null-cell lineages [2]. The clinical presentation of these conditions exhibits significant variability, ranging from the absence of symptoms to cases involving pituitary dysfunction and visual field impairment due to sellar compression. The absence of clinical symptoms results in a delay in diagnosis, with an average delay of 1.9 ± 2.9 years [1,3]. The most common symptoms associated with this condition include headache, visual field disturbance, ophthalmoplegia, and hypopituitarism. Growth patterns vary from indolent to locally aggressive with invasive behavior [4]. 

Pituitary neuroendocrine tumors (PitNETs) are low-grade and slow-growing and generally exhibit a benign behavior pattern. However, some cases may exhibit local invasion, and in rare instances, distant metastasis may occur [5]. Consequently, the 2022 WHO classification incorporates a vision that facilitates the prediction of tumor biological behavior. In this context, this study proposes a classification system based on cell lineage, which involves the identification of specific transcription factors in various cell types, including somatotropes, lactotropes, mammosomatotropes, thyrotropes, corticotropes, and gonadotropes [5]. This approach serves to complement traditional hormone immunohistochemistry techniques, which utilize antibodies against hormones such as growth hormone, prolactin, adrenocorticotropic hormone (ACTH), thyroid-stimulating hormone, follicle-stimulating hormone, and luteinizing hormone, along with the α-subunit of these hormones. Furthermore, the study proposes a subtyping system based on granulation, distinguishing between densely and sparsely granulated samples, which could serve as a marker of maturation [5]. In addition to the aforementioned subtypes, several more aggressive subtypes have been identified in PitNETs, including immature PIT1 tumors, Crooke cell tumors, silent corticotrophs, and sparsely granulated somatotrophs. Furthermore, prognostic parameters such as persistence, recurrence, and aggressiveness, including size, mitosis >2/10 HPF, local invasion, p53 positivity, and Ki67 index ≥ 3%, have been identified [5,6]. Presently, the predominant prognostic indicator is the subtype, as is also the case with other NETs [7].

NF-PitNETs exhibit heterogeneous clinical behavior, highlighting the need for reliable markers to guide surveillance, surgical decision-making, and postoperative prognostication. Reported risk factors for aggressive behavior include ACTH immunopositivity, plurihormonal or null-cell profiles, elevated Ki-67 indices (≥ 3%), and a tumor size ≥ 40 mm [6,8-11]. However, the current evidence remains inconclusive, warranting further investigation. Consequently, the primary objective of this study was to evaluate the association between the Ki-67 proliferation index and tumor size with post-surgical structural persistence in patients with NF-PitNETs. Secondarily, we aimed to provide a clinical and immunohistochemical characterization of this population within a Colombian reference center.

## Materials and methods

Data collection framework

This was a retrospective cohort study conducted by reviewing electronic medical records at Hospital de San José (Bogotá, 2010-2020) was conducted using International Classification of Diseases, Tenth Revision (ICD-10) codes related to pituitary pathology (D352, C751, D443, E240, E237, E229, E228, E221, E220, E893, E230). Data were extracted from the hospital's institutional database by two independent reviewers. It is important to note that peri-operative variables such as intraoperative blood loss, surgery duration, and detailed operative notes were not consistently documented across all cases and could not be reliably retrieved. Similarly, standardized surgical protocols were not formally recorded in a way that allows verification of uniformity across surgeons. Therefore, our analysis focuses on pre-operative and post-operative data that were consistently available.

The inclusion criteria were MRI-confirmed NF-PitNETs, surgical treatment, complete pre- and postoperative records, histopathological confirmation, and ≥12 months of follow-up. Among the 887 patients, 140 met the criteria for PitNETs, with 81 classified as non-functioning. The data collected included demographics, clinical presentation, MRI localization and size, surgical approach (transsphenoidal or transcranial), and 12-month post-operative outcomes. This subanalysis forms part of the broader PitNET surgical cohort at Hospital de San José [12].

Immunohistochemistry

For this retrospective cohort, immunohistochemical data (including Ki-67 index) were obtained from the original pathology reports and slides produced by our institutional pathology laboratory over the 10-year study period. Paraffin-embedded tissue blocks are routinely archived at our institution; however, systematic retrieval and re-staining of all blocks were not performed for this study. All immunohistochemistry was conducted according to the laboratory’s standardized internal protocols in place at the time of diagnosis.

Patients were classified according to immunohistochemical profiles as gonadotroph, prolactin, corticotroph, thyrotroph, somatotroph, plurihormonal, or null cell types. The Ki-67 labeling index was recorded as a percentage (0-100%), with ≥3% considered high on the basis of literature criteria [6,7,13-16].

Structural response

Structural persistence was defined as any radiological remnant findings after surgical resection, and disease control was defined as the absence of radiological findings after surgical management. Reoperation was defined as any surgical procedure required during follow-up. MRI follow-up was performed every six months for 12 months after surgery to determine structural persistence or healing and the need for reoperation.

Statistical analysis

Univariate analyses were performed for quantitative variables with measures of central tendency (mean and standard deviation), whereas categorical variables were reported as absolute and relative frequencies. Bivariate analyses were performed to explore associations of Ki-67 expression and tumor size with the structural persistence variable. To determine statistical significance, a two-tailed Fisher's exact test was employed, with a p-value less than 0.05 considered significant [17]. Logistic regression and chi-square analyses were not performed due to insufficient data. The statistical processing and analysis were performed via R project version 4.5.0 (R Foundation for Statistical Computing, Vienna, Austria) and R Studio.

Ethical approval

The study protocol was reviewed and approved by the ethics and research division of the University Foundation of Health Sciences SIDI6640 and was performed in accordance with the Declaration of Helsinki for biomedical research involving human patients. Written consent was obtained from SJH.

## Results

Patient demographics and clinical characteristics

A total of 140 patients were diagnosed with pituitary tumors managed with surgery, and 81 (58%) had NF-PitNETs. The mean age of the 81 patients was 51 years (SD 13.3, range 19-84), with 53% of the patients being female. Seventy-four percent of patients had macroadenomas (10-39 mm), 12% had microadenomas (<10 mm), and 13% had giant macroadenomas. In terms of structural response, 73% (59/81) of patients showed structural persistence, of which 30% (25/81) underwent reoperation. Radiotherapy was used in 12.8% (10/81) of all cases, only in patients who were not candidates for reoperation (Table 1).

**Table 1:** Characteristics of patients with NF-PitNETs. NF-PitNETs: Non-functioning pituitary neuroendocrine tumors

Variables	n (%), total = 81
Age, years; mean (SD)	51.8 (13.3)
Female, n(%)	43 (53%)
Weight, kg (SD)	78,20 (16.78)
Tumor size at diagnosis, n(%)	
<10 mm	10 (12.34%)
10-39 mm	60 (74.07%)
≥ 40 mm	11 (13.58%)
Surgical approach, n(%)	
Transsphenoidal	65 (80.24%)
Transcranial	16 (19.75%)
Radiotherapy, n(%)	10 (12.34%)
Structural response, n(%)	
Incomplete	59 (72.83%)
Complete	22 (27.16%)
Surgical reintervention, n(%)	
Yes	25 (30.86%)
No	56 (69.13%)

The most prevalent symptom was visual field disorder, which was observed in 70% of the patients, followed by headache, which was observed in 66% of cases. The prevalence of menstrual cycle disorders was observed in 13% of the female population (6/43), whereas other less prevalent symptoms included erectile dysfunction in male patients (7%; 3/38), galactorrhoea (7%), decreased libido (5%), and infertility (1%). As indicated by the medical history, obesity was observed in 34% of the samples, high blood pressure in 24%, dyslipidemia in 12%, diabetes mellitus in 8.6%, carbohydrate intolerance in 6%, and major cardiovascular disease in 1%.

Immunohistochemistry

**Figure 1:**
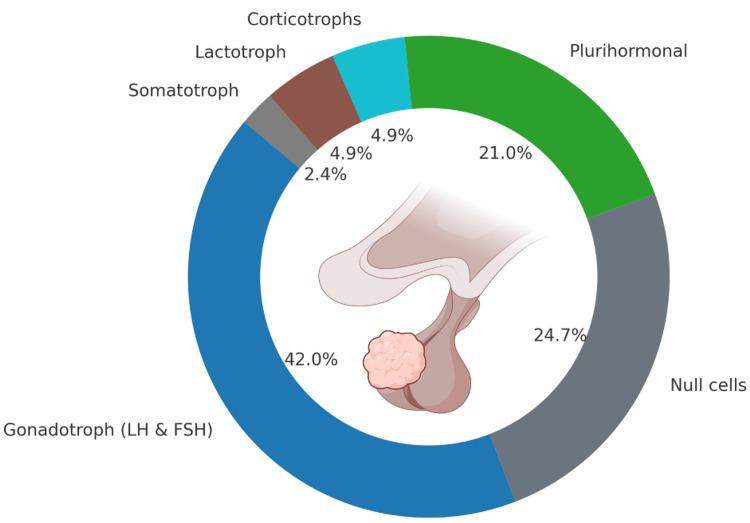
Distribution of immunoreactivity in NF-PitNETs. NF-PitNETs: Non-functioning pituitary neuroendocrine tumors Created in BioRender by Jimenez Canizales, C. (2025) https://BioRender.com/wbxil3z

Immunoreactivity analysis revealed that gonadotrophs were the most prevalent (42%), followed by null cells (24%), corticotrophs (4.9%), and lactotrophs (4.9%) (Figure 1). Ki-67 indices were available for 67 patients, of whom 78% had values ≥3% and 22% had values <3%.

Ki-67 index and tumor size analysis

The study revealed that patients with a Ki-67 index ≥ 3% had a 100% incidence of structural persistence, while those with a Ki-67 index <3% had a 72% incidence. This difference was statistically significant (p = 0.028, Fisher's test). With respect to tumor size, 100% (15/15) of patients with a size ≥ 40 mm exhibited structural persistence, whereas 68% (48/70) of patients with a size < 40 mm and 50% (5/10) of those with a size < 10 mm demonstrated structural persistence (p = 0.030, Fisher's test). The characteristics according to the Ki-67 index are described in Table 2.

**Table 2:** Clinical and immunoreactivity characteristics in patients with NF-PitNETs according to the Ki-67 index. NF-PitNETs: Non-functioning pituitary neuroendocrine tumors *Fisher test and the significant p-value was 0.05

	Ki-67% index < 3% (n=52)	Ki-67% index ≥ 3% (n=15)	p-value*
Tumor size at diagnosis			1
<10 mm	6	3	
10-39 mm	39	10	
>40 mm	7	2	
Immunoreactivity			0.701
Gonadothrops	22	7	
Lactotrophs	3	0	
Tirotrophs	0	0	
Somatotrophs	1	1	
Corticothrophs	2	1	
Plurihormonals	12	2	
Nulls cells	12	4	
Structural response			0.028
Complete	15	0	
Incomplete	37	15	
Surgical reintervention	15	8	0.121
Radiotherapy	3	6	0.002
Surgical approach			0.146
Transcraneal	8	5	
Transsphenoidal	44	10	

## Discussion

We searched the PubMed, Scopus, and SciELO databases and this study represents one of the largest surgical cohorts of NF-PitNETs in Colombia [12,18]. A structural response was achieved in 73% of the patients, whereas a tumor size ≥40 mm and Ki-67 ≥3% emerged association variable of structural persistence. Ki-67, a nuclear antigen expressed in all active phases of the cell cycle except G0, is a widely recognized proliferation marker in multiple tumors, including NF-PitNETs. Although the aggressiveness threshold varies, values ≥3% have been linked to increased invasiveness and recurrence risk [7]. Notably, 67% of incomplete resections with postoperative regrowth in prior studies also showed elevated Ki-67 [6,11,16,19,20]. However, other studies have not demonstrated an association [15], and some consensus guidelines employ considerably greater aggressiveness [14].

With respect to immunoreactivity, the most prevalent cell type in our study was gonadotropes, followed by null cells. These findings are consistent with the results of another study, which reported that 58.1% of cases corresponded to gonadotropes, whereas 23% of tumors were negative for hormonal markers and 9.9% were silent corticotropic adenomas [21].

As reported in the literature, the clinical manifestations of non-functioning pituitary tumors appear late and may not be diagnosed until they cause a mass effect on adjacent structures, causing symptoms such as visual disturbances, which are the most frequent symptoms in 67% of cases, and headache, which has been reported in 19-75% of cases depending on size [22-24]. The results of this study are consistent with those reported in the extant literature, which also identified visual field disorders as the primary symptom, followed by headache and, less frequently, manifestations of hormonal disturbances such as menstrual cycle disorders, erectile dysfunction, galactorrhoea, and decreased libido [24].

Limitations

Retrospective Data Availability

A significant limitation of this study is its retrospective design, which constrained our ability to obtain granular perioperative data. Variables such as the extent of intraoperative resection, surgical technique, and surgeon-specific decision-making processes were not uniformly documented in the patient records over the 10-year span. Consequently, we could not assess the impact of surgical variability on outcomes. Future prospective studies should implement standardized documentation protocols to capture these critical variables.

Immunohistochemical Variability

A major limitation of this study is the potential variability in immunohistochemical procedures over the 10-year inclusion period. Because this was a retrospective analysis, we relied primarily on the immunohistochemistry performed at the time of diagnosis and did not systematically re-stain all archived tissue blocks. Furthermore, the Ki-67 index value was not confirmed by digital pathology. Therefore, despite the use of standardized institutional protocols, full consistency and uniformity of Ki-67 assessment and other immunohistochemical markers across the entire cohort cannot be guaranteed. This constraint should be taken into account when interpreting the proposed cut-off values and their reproducibility in other settings. We acknowledge that a prospective study with a rigorously defined and consistently applied IHC protocol is necessary to validate our results and establish a universal cut-off value [5,24].

Molecular Classification and Transcription Factor Analysis

While we acknowledge that transcription factor immunohistochemistry (SF1, TPIT, PIT1) was not performed, the implications of this omission warrant deeper discussion. The absence of these markers precludes definitive lineage determination and may lead to misclassification of true adenohypophyseal cell types, particularly mislabeling transcription-factor-positive tumors as "null-cell" adenomas. This diagnostic imprecision could affect the generalizability of our Ki-67 and tumor size thresholds, as different pituitary lineages exhibit distinct biological behaviors [5,24]. Our findings should therefore be interpreted as applicable to a "real-world" NF-PitNET cohort where complete molecular profiling is unavailable, a common scenario in many institutions. Prospective validation with full transcription factor panels is essential to determine whether our prognostic markers retain significance within specific adenohypophyseal lineages.

Statistical Analysis and Causal Inference

Our reliance on univariate and bivariate statistical testing, without multivariate adjustment, represents a significant methodological constraint that limits causal inference. While we identified significant associations between Ki-67, tumor size, and structural persistence, we could not control for potential confounders such as surgeon experience, subtle variations in surgical technique, or patient-specific anatomical challenges. A multivariate model would have allowed us to assess the independent predictive value of each marker. However, due to our sample size and the retrospective availability of covariates, multivariate analysis was not feasible. Consequently, our results should be considered hypothesis-generating rather than definitive predictive tools.

Surgical and Postoperative Variability

The retrospective nature of our study prevented a detailed assessment of surgical decision-making variability. Similarly, postoperative management, including criteria for early adjuvant therapy (radiation or medical treatment) and the intensity of radiological follow-up, was not standardized and may have influenced long-term outcomes. This unmeasured variability could confound the relationship between tumor characteristics and persistence and represents a critical area for prospective standardization in future research.

In contrast, the strategy used to delineate the structural response of the tumor lacks the ability to distinguish between tumor regrowth and structural persistence, a distinction that becomes relevant when the Ki-67 index is interpreted as a marker of cell proliferation [7]. A notable limitation was the absence of detailed radiological characteristics of the pituitary lesions.

Implications for future research

Our study, despite its retrospective nature and the inherent variability in a 10-year span of immunohistochemical staining, provides a foundational dataset that can inform the design of more robust, prospective investigations. The significant association we identified between Ki-67 ≥3% and tumor size ≥40 mm with post-surgical structural persistence offers a clear, testable hypothesis for future work. To enhance reproducibility and comparability, prospective studies should implement a standardized IHC protocol for Ki-67 quantification from the outset, ideally incorporating digital image analysis to minimize inter-observer variability. Furthermore, future research should expand beyond Ki-67 to include a full panel of transcription factors (SF1, TPIT, PIT1) and other proliferation markers (mitotic count, p53) as per the 2022 WHO classification, which will allow for a more precise subtyping of NF-PitNETs and validation of our findings within specific lineages. Multicenter studies are required to assemble larger cohorts, which would enable the development and validation of a predictive model integrating clinical, radiological, and molecular data.

## Conclusions

This 10-year retrospective cohort study provides a comprehensive characterization of NF-PitNETs in a Latin American tertiary center, revealing a population predominantly affected by macroadenomas (89%) with a high prevalence of preoperative visual deficits (70%). Our primary finding is that both a Ki-67 proliferation index ≥3% and a maximum tumor diameter ≥40 mm are significantly associated with post-surgical structural persistence (p=0.028 and p=0.030, respectively). We also identified a substantial rate of pituitary insufficiency post-surgery and a notable proportion of plurihormonal tumors, underscoring the complex endocrine landscape of this cohort. However, the retrospective design, dynamic changes in data recording over time, and lack of transcription factor analysis impose significant constraints on data interpretation and generalizability. Consequently, these markers show promise as potential tools for risk stratification in resource-limited settings but should be interpreted cautiously until prospective validation confirms their predictive utility. Future research should build upon this work by implementing prospective data collection, standardized immunohistochemistry with transcription factor panels, and multivariate modeling to validate and refine these thresholds. Our detailed reporting of methodological challenges aims to guide fellow researchers in designing more robust studies, particularly in resource-limited settings where such retrospective analyses remain a necessary first step.
